# Supplementary material for: Metabolomics and Transcriptomics Provide Insights into Lipid Biosynthesis in the Embryos of Walnut (Juglans regia L.)
Source: Plants (Basel). 2023 Jan 24;12(3):538. doi: 10.3390/plants12030538 (PMC9921657; doi:10.3390/plants12030538)
Supplement: Supplementary file 1 [file plants-12-00538-s001.zip › Figure S1.pdf]

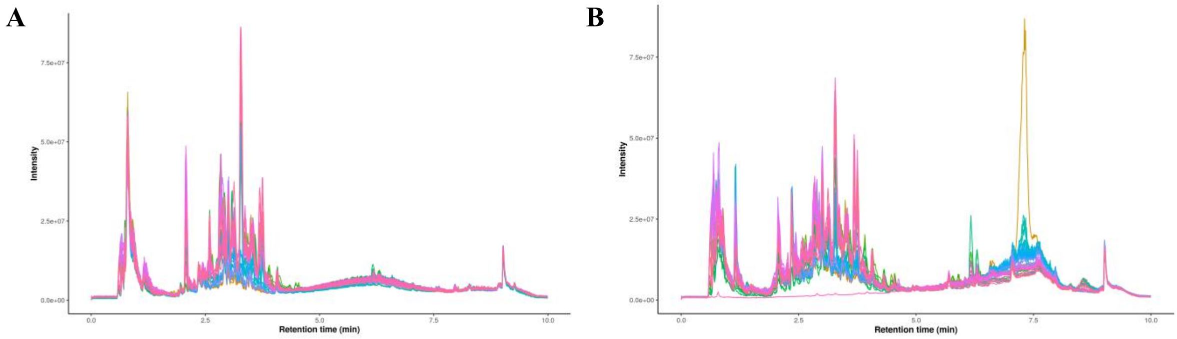

Figure S1. Total ion chromatogram. **(A)** Total ion chromatogram in NEG model. **(B)** Total ion chromatogram in POS model. The x-axis represents retention time and the y-axis represents the sum of intensity for all the ions in MS. The overlaps of QC samples in TIC can be used to preliminarily judge the state of the instrument. The higher intensity overlap presents, the more stable the instrument is.
